# Phosphocholine-Modified Lipooligosaccharides of *Haemophilus influenzae* Inhibit ATP-Induced IL-1β Release by Pulmonary Epithelial Cells

**DOI:** 10.3390/molecules23081979

**Published:** 2018-08-08

**Authors:** Katrin Richter, Christian Koch, Alexander Perniss, Philipp M. Wolf, Elke K. H. Schweda, Sven Wichmann, Sigrid Wilker, Ilona Magel, Michael Sander, J. Michael McIntosh, Winfried Padberg, Veronika Grau

**Affiliations:** 1 Laboratory of Experimental Surgery, Department of General and Thoracic Surgery, Justus-Liebig-University Giessen, German Center for Lung Research, 35392 Giessen, Germany; Philipp.M.Wolf@bio.uni-giessen.de (P.M.W.); sigridwilker@web.de (S.W.); Winfried.Padberg@chiru.med.uni-giessen.de (W.P.); Veronika.Grau@chiru.med.uni-giessen.de (V.G.); 2 Department of Anesthesiology and Intensive Care Medicine, Justus-Liebig-University Giessen, 35392 Giessen, Germany; christian.koch@chiru.med.uni-giessen.de (C.K.); Sven.Wichmann@outlook.de (S.W.); Ilona.Magel@chiru.med.uni-giessen.de (I.M.); Michael.Sander@chiru.med.uni-giessen.de (M.S.); 3 Institute of Anatomy and Cell Biology, Justus-Liebig-University Giessen, German Center for Lung Research, 35385 Giessen, Germany; Alexander.Perniss@anatomie.med.uni-giessen.de; 4 Division of Chemistry, Department of Physics, Chemistry and Biology, Linköping University, S-58183 Linköping, Sweden; elke.schweda@liu.se; 5 Department of Biology, University of Utah, Salt Lake City, UT 84112, USA; mcintosh.mike@gmail.com; 6 George E. Wahlen Veterans Affairs Medical Center, Salt Lake City, UT 84148, USA; 7 Department of Psychiatry, University of Utah, Salt Lake City, UT 84108, USA

**Keywords:** A549, Calu-3, CHRNA7, CHRNA9, CHRNA10, immune evasion, inflammasome, lung, nicotinic acetylcholine receptor, phosphocholine-modification

## Abstract

Phosphocholine-modified bacterial cell wall components are virulence factors enabling immune evasion and permanent colonization of the mammalian host, by mechanisms that are poorly understood. Recently, we demonstrated that free phosphocholine (PC) and PC-modified lipooligosaccharides (PC-LOS) from *Haemophilus influenzae*, an opportunistic pathogen of the upper and lower airways, function as unconventional nicotinic agonists and efficiently inhibit the ATP-induced release of monocytic IL-1β. We hypothesize that *H. influenzae* PC-LOS exert similar effects on pulmonary epithelial cells and on the complex lung tissue. The human lung carcinoma-derived epithelial cell lines A549 and Calu-3 were primed with lipopolysaccharide from *Escherichia coli* followed by stimulation with ATP in the presence or absence of PC or PC-LOS or LOS devoid of PC. The involvement of nicotinic acetylcholine receptors was tested using specific antagonists. We demonstrate that PC and PC-LOS efficiently inhibit ATP-mediated IL-1β release by A549 and Calu-3 cells via nicotinic acetylcholine receptors containing subunits α7, α9, and/or α10. Primed precision-cut lung slices behaved similarly. We conclude that *H. influenzae* hijacked an endogenous anti-inflammatory cholinergic control mechanism of the lung to evade innate immune responses of the host. These findings may pave the way towards a host-centered antibiotic treatment of chronic airway infections with *H. influenzae*.

## 1. Introduction

Strains of *Haemophilus influenzae* can be divided into two categories: the encapsulated, typeable strains and the genetically highly variable non-encapsulated, non-typeable strains (NTHi) [1,2]. A well-described virulence factor of most wild-type NTHi is the *lic1* operon that encodes enzymes needed for the synthesis of phosphocholine-modified lipooligosaccharides (PC-LOS) [3,4,5,6,7,8,9]. *H. influenzae* strains carrying a non-functional mutant *lic1*-operon are rapidly cleared from the airways of experimental mice and rats, whereas PC-LOS-positive wild-type strains are selected and can cause severe pulmonary infections [6,10]. Accordingly, most human respiratory *H. influenzae* infections are due to strains with a functional *lic1*-operon [8]. Like *H. influenzae*, numerous Gram-negative and Gram-positive bacteria as well as eukaryotic parasites produce PC-modified cell surfaces or secretory products that are generally regarded as immunomodulatory mediators enabling pathogen survival [7,8].

PC-LOS from wild-type *H. influenzae* strains is at best a weak inducer of costimulatory molecules CD40 and CD58 as well as interleukin-1β (IL-1β) and tumor necrosis factor-α (TNF-α) mRNA in human monocytic THP-1 cells, whereas PC-free LOS from a *lic1*-mutant is as efficient as lipopolysaccharide (LPS) from *Escherichia coli* [11]. It is, however, unclear if PC-LOS only weakly activates Toll-like receptor 4 or if other mechanisms are involved that control the expression and release of pro-inflammatory cytokines including IL-1β. A better understanding of immune evasion strategies is needed for the development of novel anti-infective therapies to treat *H. influenzae* infections.

IL-1β is a highly potent pro-inflammatory cytokine of innate immunity that plays an essential role in host defense against infections [12,13]. As excessive systemic IL-1β levels can cause fever, shock and multiple organ failure, including acute respiratory distress syndrome [13,14,15], a tight regulation of its release is vital. The production of IL-1β often requires two consecutive so-called “danger signals” [13,16,17]. The pathogen-associated molecular pattern LPS is a typical first signal inducing the biosynthesis of cytoplasmic pro-IL-1β, an inactive cytoplasmic pro-form of IL-1β. Extracellular ATP is an indicator of severe cellular damage and a prototypical second danger signal that initiates ion currents at P2X7 receptors, including efflux of potassium ions. Reduced cytoplasmic potassium levels leads to the assembly of the NACHT, LRR and PYD domains-containing protein 3 (NLRP3)-containing inflammasome and to caspase-1 activation [13,16,17]. Caspase-1 enables the rapid maturation and release of cytokines of the IL-1 family including IL-1β [13,16,17].

We recently reported that agonists of nicotinic acetylcholine receptors (nAChRs) containing subunits α7, α9 and/or α10 efficiently inhibit the ATP-induced release of IL-1β by human monocytic cells [18,19,20]. Apart from classical nicotinic agonists such as acetylcholine (ACh), choline or nicotine, free PC and PC-LOS from bacterial walls of wild-type *H. influenzae* function as unconventional nAChR agonists that also inhibit the ATP-mediated IL-1β release [18,19,20]. In contrast, PC-free LOS isolated from *H. influenzae lic1*-mutants is ineffective [18]. Interestingly, the covalent binding of PC to LOS enhances the efficiency of PC by a factor of about 400 [18].

Up to now, we investigated the inhibition of ATP-induced IL-1β by nicotine, PC and PC-LOS in human monocytic blood cells [18]. In addition to mononuclear phagocytes of the lung, respiratory and alveolar epithelial cells contribute to host defense against respiratory infections by NLRP3 inflammasome activation and IL-1β release [15,21,22,23,24,25,26]. As those epithelial cells are among the first cells that come into contact with pathogens, they are expected to be of particular importance during the early phase of infection. Here, we confirm our hypothesis that nicotine, PC and PC-LOS inhibit the ATP-induced release of IL-β by human cancer cell lines A549, resembling alveolar epithelial cells type II [27,28], and Calu-3 cells, an established model for bronchial epithelial cells [29]. PC-LOS is also active in mouse lung tissue. Moreover, we provide evidence that nicotine, PC and PC-LOS signal via epithelial nAChR subunits α7 and α9, while PC-free LOS isolated from *lic*-mutants is inactive.

## 2. Results

### 2.1. Nicotine and PC Inhibit IL-1β Release from A549 Cells via nAChRs

**Figure 1:**
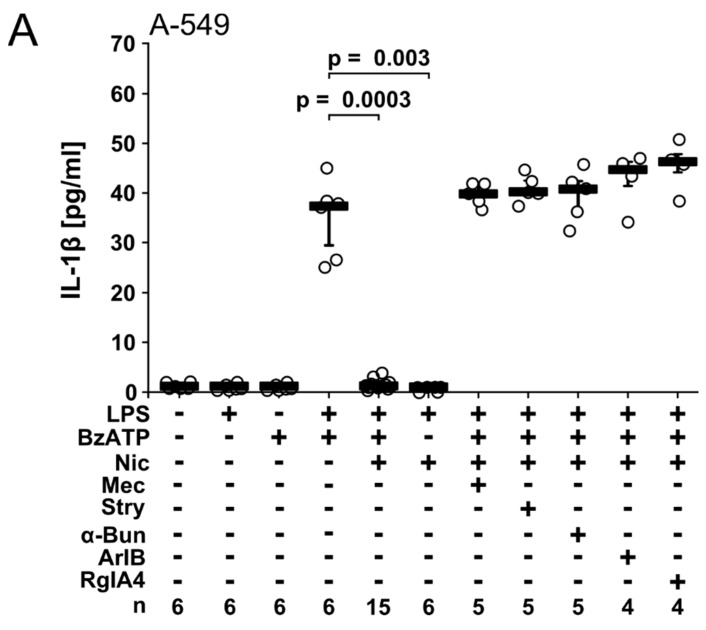

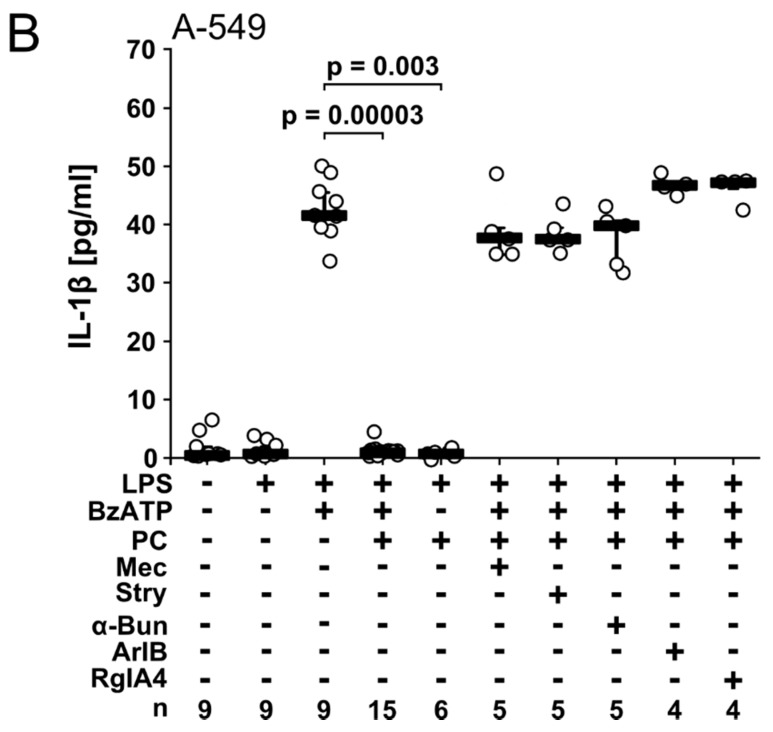
Nicotine (Nic) and phosphocholine (PC) inhibit the release of IL-1β by A549 cells. Human LPS-primed A549 cells were stimulated with 2′(3′)-O-(4-benzoylbenzoyl)adenosine-5′-triphosphate (BzATP, 100 µM) in the presence or absence of Nic (100 µM) (**A**) or PC (100 µM) (**B**) and the IL-1β released to the supernatant was measured 30 min later. The inhibitory effects of Nic and PC were sensitive to nicotinic antagonists mecamylamine (Mec; 100 µM), strychnine (Stry; 10 µM), α-bungarotoxin (α-Bun; 1 µM), [V11L, V16D]ArIB (500 nM), or RgIA4 (200 nM). Data are presented as individual data points, bars represent median, whiskers encompass the 25th to 75th percentile; n-numbers of independent experiments are indicated in the figure. Experimental groups were compared by Kruskal Wallis test followed by Mann Whitney rank sum test.

We reported before that nicotine and PC inhibit the ATP-induced IL-1β release by human monocytic cells via nAChRs containing subunits α7, α9 and α10 [18,19,20]. To test whether this applies to lung epithelial cells, we primed A549 cells with LPS from *E. coli* (100 ng/mL) for 24 h followed by stimulation with the P2X7 receptor agonist 2′(3′)-O-(4-benzoylbenzoyl)adenosine 5′-triphosphate trieethylammonium salt (BzATP, 100 µM) for another 30 min. IL-1β released into the cell culture supernatant was measured by enzyme-linked immunosorbent assay (ELISA) (Figure 1A,B). The concentration of IL-1β in the cell culture supernatant ranged between 25 and 50 pg/mL. When either priming with LPS or stimulation with BzATP was omitted, virtually no IL-1β was detected (Figure 1A,B). Nicotine (100 µM; *p* = 0.000, *n* = 15; Figure 1A) and PC (100 µM, *p* = 0.0001, *n* = 15; Figure 1B) fully inhibited the BzATP-induced release of IL-1β from LPS-primed A549 cells. In control experiments, in which nicotine or PC were added to LPS-primed cells in the absence of BzATP, virtually no IL-1β was released (Figure 1A,B). Cell viability as estimated by the measurement of the cytoplasmic enzyme lactate dehydrogenase (LDH) in cell culture supernatants was unimpaired in these and in all following experiments.

To analyze if nicotine and PC signal via nAChRs in A549 cells, a panel of different nAChR antagonists was used: (1) mecamylamine (100 µM), a general nAChR antagonist, (2) α-bungarotoxin (1 µM), an antagonist targeting nAChRs containing subunits α7 or α9, (3) strychnine (10 µM) that preferentially antagonizes nAChRs containing subunit α9, (4) α-conotoxin [V11L, V16D]ArIB (500 nM), a specific antagonist of nAChR α7, and 5) α-conotoxin RgIA4 (200 nM) that antagonizes nAChRs containing subunits α9/α10 [19,30,31,32,33,34]. When applied together with BzATP, all nAChR antagonists abolished the inhibitory effect of nicotine and PC and enabled the full release of IL-1β (Figure 1A,B). In control experiments, where BzATP was omitted, none of the nAChR antagonists induced the release of IL-1β by LPS-primed A549 cells (data not shown). We conclude from these data that nicotine and PC are efficient inhibitors of BzATP-induced IL-1β release by pulmonary epithelial cells, and that nAChR subunits α7, α9 and/or α10 are involved in signaling of nicotine and PC.

### 2.2. Nicotine and PC Inhibit IL-1β Release from Calu-3 Cells via nAChRs

**Figure 2:**
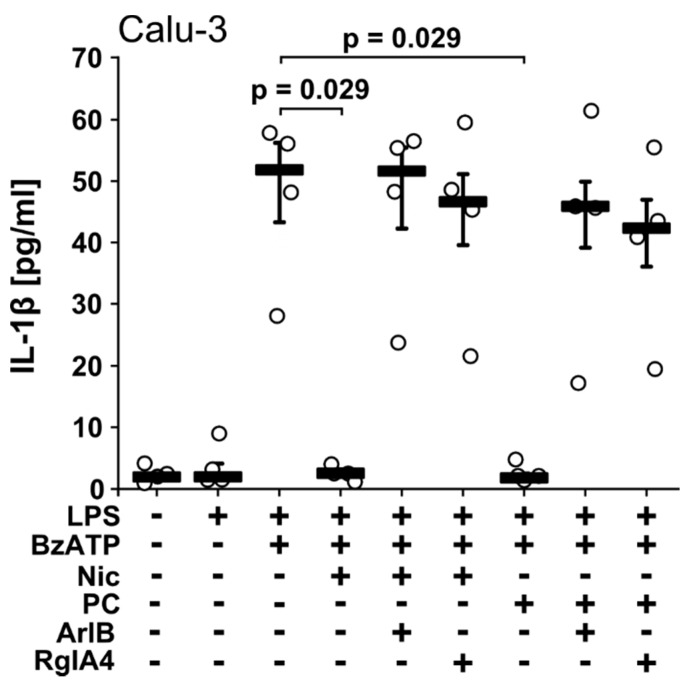
Nicotine (Nic) and phosphocholine (PC) inhibit the release of IL-1β by Calu-3 cells. Human LPS-primed Calu-3 cells were stimulated with BzATP (100 µM) in the presence or absence of Nic (100 µM) or PC (100 µM). [V11L, V16D]ArIB (500 nM) or RgIA4 (200 nM) was added together with BzATP and IL-1β released to the supernatant was measured after 30 min. In one of the experiments, IL-1β levels released in response to BzATP was low throughout. Data are presented as individual data points, bars represent median, whiskers encompass the 25th to 75th percentile, *n* = 4 per experiment. Experimental groups were compared by Kruskal Wallis test followed by Mann Whitney rank sum test.

In the next set of experiments, we demonstrated that Calu-3 cells essentially reacted like A549 cells (Figure 2). Application of BzATP (100 µM) to LPS-primed Calu-3 cells resulted in the release of IL-1β into the cell culture supernatant with concentrations ranging between 28 and 58 pg/mL (Figure 2) that was fully inhibited in the presence of nicotine (100 µM; *p* = 0.029, *n* = 4; Figure 2) or PC (100 µM, *p* = 0.029, *n* = 4; Figure 2). These inhibitory effects were fully reversed by addition of [V11L, V16D] ArIB (500 nM) or RgIA4 (200 nM; Figure 2), suggesting that also in Calu-3 cells nAChR subunits α7, α9 and/or α10 are involved in signaling of nicotine and PC [18].

### 2.3. PC-LOS Inhibit BzATP-Mediated IL-1β Release from A549 and Calu-3 Cells

**Figure 3:**
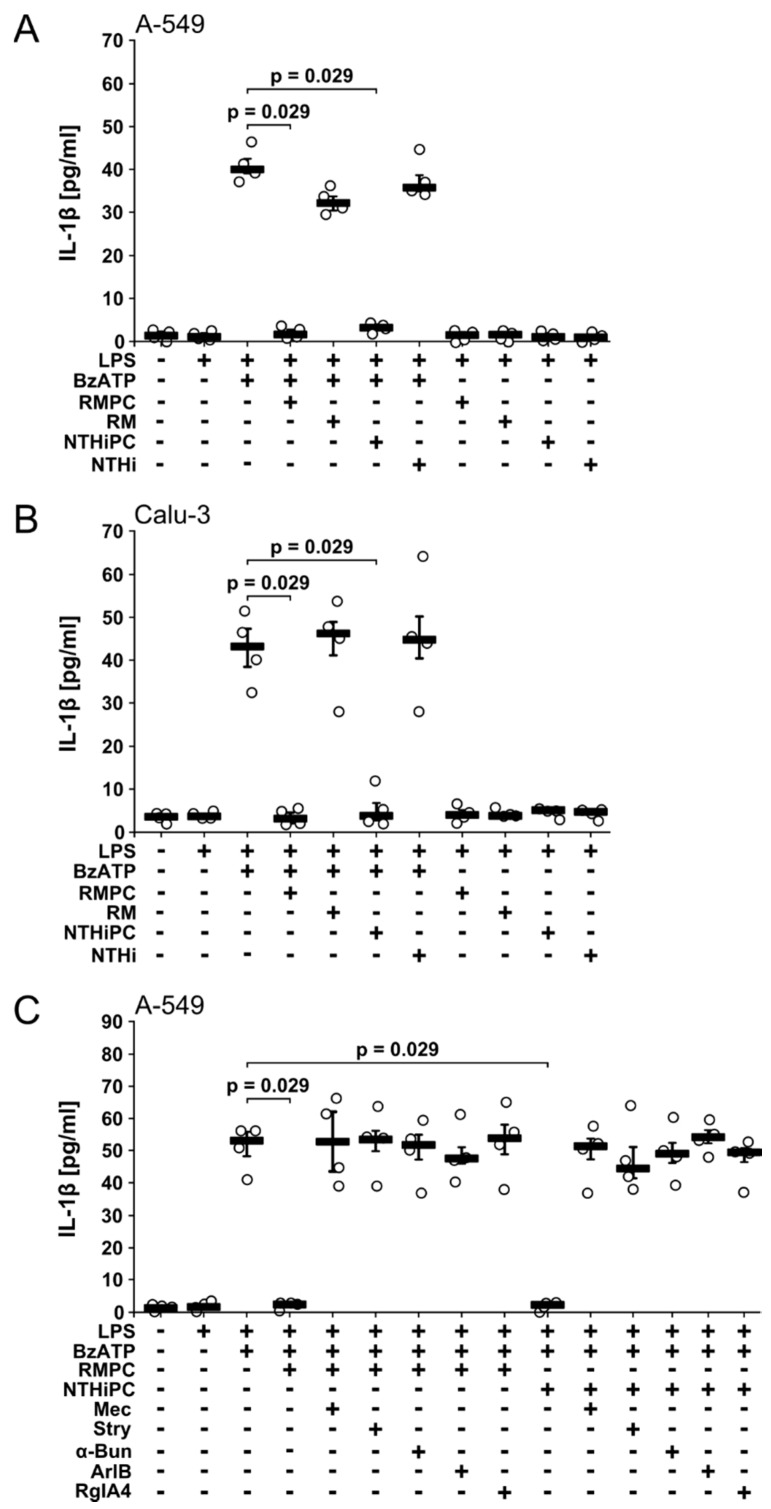
Phosphocholine-modified lipooligosaccharides (PC-LOS) isolated from *H. influenzae* strains inhibit the release of IL-1β by A549 and Calu-3 cells. Human LPS-primed A549 (**A**,**C**) or Calu-3 (**B**) cells were stimulated with BzATP (100 µM) in the presence or absence of PC-LOS isolated from the *H. influenzae* strains RM118 (RMPC; 1 µg/mL) and NTHi 1233 (NTHiPC; 1 µg/mL) and IL-1β released to the supernatant was measured after 30 min. PC-free LOS from the corresponding *lic1*-mutant strains RM7004-*lic1* (RM; 1 µg/mL) and NTHi1233-*lic1* (NTHi; 1 µg/mL) lacking the PC-modification were included as a negative control. (**C**) The inhibitory effects of RMPC and NTHiPC were reversed by nicotinic antagonists mecamylamine (Mec; 100 µM)), strychnine (Stry; 10 µM), α-bungarotoxin (α-Bun; 1 µM), [V11L, V16D]ArIB (500 nM), or RgIA4 (200 nM). Data are presented as individual data points, bars represent median, whiskers encompass the 25th to 75th percentile, *n* = 4 per experiment. Experimental groups were compared by Kruskal Wallis test followed by Mann Whitney rank sum test.

We showed before that nicotine, PC and PC-LOS inhibit the ATP-induced release of IL-1β from monocytic cells via nAChR subunits α7 and α9, while PC-free LOS isolated from *lic1*-mutants is inactive [18]. Here, we tested the effects of PC-LOS from two independent *H. influenzae* strains, RM118 and NTHi123323 in the epithelial cell lines A549 and Calu-3. PC-LOS (1 µg/mL) purified from both strains efficiently inhibited the BzATP-induced release of IL-1β from LPS-primed A549 and Calu-3 cells (Figure 3A,B). The concentration of PC-LOS used in this study has been determined before [18]. In contrast, PC-free LOS from the corresponding mutant strains lacking the PC-modification, RM7004-*lic1* and NTHi1233-*lic1* [4], was ineffective (Figure 3A,B). We selected A549 cells to test the hypothesis that PC-LOS signals via the same nAChR subunits like free PC. The inhibitory effects of PC-LOS were sensitive to mecamylamine (100 µM), α-bungarotoxin (1 µM), strychnine (10 µM), [V11L, V16D]ArIB (500 nM), and RgIA4 (200 nM) (Figure 3C), suggesting that PC-LOS signals via nAChR subunits α7, α9 and/or α10 similar to free PC.

### 2.4. PC-LOS Inhibit the BzATP-Mediated IL-1β Release from Mouse Precision Cut Lung Slices (PCLS)

**Figure 4:**
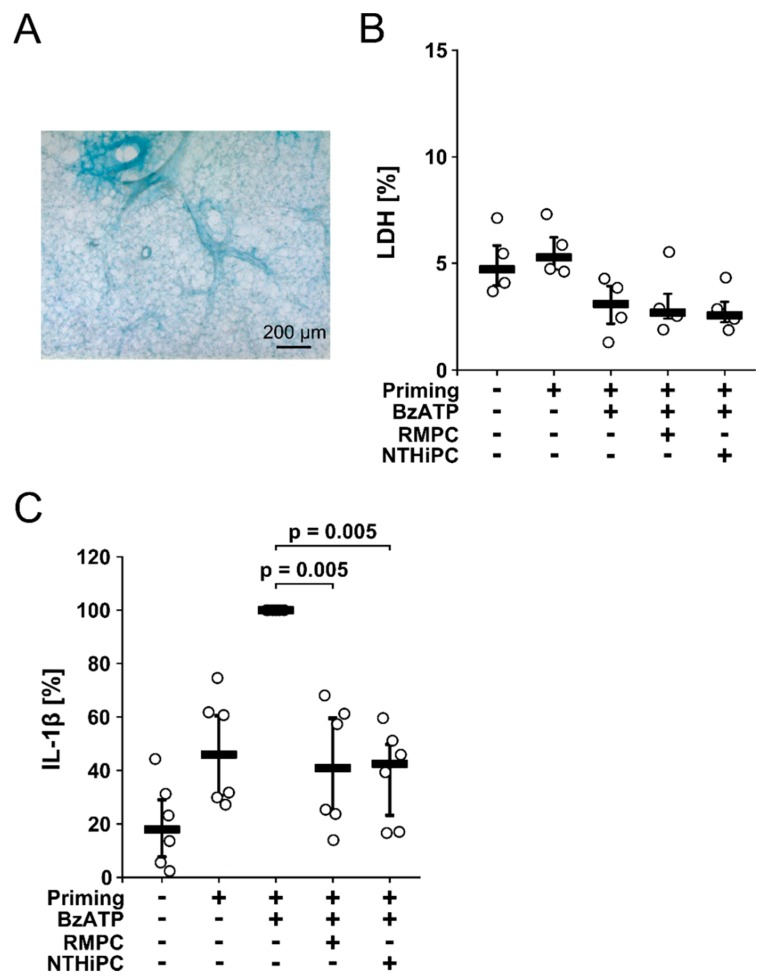
Phosphocholine-modified lipooligosaccharides (PC-LOS) isolated from *H. influenzae* strains inhibit the release of IL-1β by wild-type mouse precision cut lung slices (PCLS, *n* = 6). (**A**) A fixed PCLS, lightly stained with Richardson’s stain depicts normal pulmonary morphology; (**B**,**C**) PCLS were primed with LPS (100 ng/mL), IFN-ϒ (20 ng/mL) and TNF-α (10 ng/mL) for 24 h followed by application of BzATP (150 µM) in the presence or absence of PC-LOS isolated from the *H. influenzae* strains RM118 (RMPC; 1 µg/mL) and NTHi 1233 (NTHiPC; 1 µg/mL); (**B**) Lactate dehydrogenase (LDH) was measured in the cell culture supernatant 30 min later and expressed as % of the total LDH content of the individual PCLS (*n* = 4). Due to a technical problem, LDH release was not measured in two out of six experiments; (**C**) IL-1β was measured in cell culture supernatant at the same time point as the LDH (*n* = 6). The IL-1β concentration in experiments where primed PCLS were stimulated with BzATP was set to 100% and all other values were calculated accordingly. (**B**,**C**) Data are presented as individual data points, bars represent median, whiskers encompass the 25th to 75th percentile. Experimental groups were compared by the Wilcoxon signed-rank test.

To test, if the inhibitory potential of PC-LOS on the BzATP-induced release of IL-1β also applies to lung tissue, we investigated PCLS from healthy wild-type mice, an established ex vivo model to investigate pulmonary inflammation [35]. PCLS were cultured for 48 h. The lung tissue retained normal morphology (Figure 4A) and viability as estimated by the release of LDH (Figure 4B).

Thereafter, PCLS were primed with LPS (100 ng/mL), interferon-γ (IFN-γ; 20 ng/mL) and TNF-α (10 ng/mL) for 24 h and BzATP (100 µM) was added to the tissue culture supernatant for 30 min in the presence or absence of PC-LOS (1 µg/mL) purified from *H. influenzae* strains RM118 or NTHi123323. During priming, PCLS released IL-1β into the tissue culture supernatant (4–28 pg/mg, *n* = 6; Figure 4B), which was further increased by addition of BzATP (13–46 pg/mg, *n* = 6; Figure 4B). PC-LOS fully inhibited the BzATP-induced IL-1β release (RM118: 3–28 pg/mg, *p* = 0.005, *n* = 6; NTHi123323: 2–27 pg/mg, *p* = 0.005, *n* = 6; Figure 4B) suggesting that the inhibitory potential of PC-LOS also applies to the complex lung tissue.

## 3. Discussion

We demonstrate here that the ATP-induced release of IL-1β by the pulmonary epithelial cell lines A549 and Calu-3 is efficiently inhibited by nicotine, PC and PC-LOS isolated from wild-type *H. influenzae* bacterial cell walls, whereas PC-free LOS isolated from mutant stains is inactive. We further show that PC-LOS exerts similar effects in PCLS, an ex vivo model that is suited to investigate aspects of pulmonary inflammation [35,36,37]. In addition, we are the first to demonstrate that PC and PC-LOS function as unconventional nAChR agonists signaling via nAChR subunits α7, α9 and/or α10 in pulmonary epithelial cells. We suggest that this mechanism contributes to the known PC-LOS-mediated immune evasion of *H. influenzae* [6,7,8,9].

Several mechanisms have been suggested to be involved in the positive selection of PC-LOS-exposing *H. influenzae* strains on epithelial surfaces, where NTHi strains are predominantly found. These mechanisms include molecular mimicry, increased adherence of PC-LOS-exposing bacteria to respiratory epithelia by binding to the receptor of the platelet activating factor (PAF) [38,39] as well as PC-LOS-dependent biofilm formation that facilitates host colonization [40,41,42] and protection from the attack of host proteases [43].

The dominance of the PC-LOS-carrying NTHi variants in vivo is surprising in the light of known host effector mechanisms targeting PC-LOS. The acute-phase reactant C-reactive protein (CRP) binds to cell surface-exposed PC-LOS, and is expected to fix complement and to result in pathogen elimination [10,44,45,46]. In addition, high titers of natural antibodies to PC are ubiquitously found in humans [47,48]. They should also enable a quick eradication of PC-LOS exposing *H. influenzae* [49,50,51,52]. These anti-bacterial attack mechanisms, however, are expected to be predominantly active in the blood plasma, where *H. influenzae* strains lacking the PC-decoration on their cell wall might have a better chance of survival [10]. In contrast, PC-LOS-positive strains seem to have a survival benefit in the respiratory tract, the source of most PC-LOS-positive *H. influenzae* isolates from infected patients [8]. Accordingly, frequent spontaneous phase variations have been observed regarding the on-off switching in the exposition of PC-LOS on the bacterial surface [53], a mechanism that might enable a quick adaptation of *H. influenzae* to its environment within the host. These considerations prompted us to focus on respiratory and pulmonary epithelial cells.

Epithelial cells including lung epithelial cells form the first line of mechanical and immunological defense against infections. In response to numerous microbial noxes and sterile cell damage leading to the release of cytoplasm, inflammasomes assemble and exert vital functions regarding the preservation of the epithelial integrity and in the coordination of the immune response [15,21,24,25,26]. In vivo analyses and ex vivo experiments on intact tissues are difficult to interpret regarding the origin of released mediators, because they contain a multitude of different cell types including leukocytes. We used A549 cells resembling alveolar epithelial cells type II [27,28], and the bronchial epithelial cell line Calu-3 [29] to ascertain the purity of the epithelial cells. The capacity A549 cells to form NLRP3 inflammasomes and to secrete mature IL-1β has been shown before [54,55] and we demonstrate in this study that Calu-3 cells are also able to secrete IL-1β. These data must be interpreted with caution, because cell lines never fully reflect the properties of primary cells. In future experiments, A549 cells, Calu-3, or primary lung epithelial cells should be investigated in air liquid interface cultures that more closely mimic many of the features of the native polarized airway epithelium.

To approach reality, we investigated PCLS, thin slices of the highly complex lung tissue. In this setting, the cellular source of IL-1β released by PCLS is unclear because the lung tissue is composed of numerous cell types including monocytes and macrophages. However, PC-LOS was able to fully inhibit the IL-1β release caused by stimulation with BzATP, which is in line with our hypothesis. PCLS are widely used to study the lung ex vivo, to reduce the number of experiments on living animals (e.g., [35,56,57]). This approach is not without controversy because PCLS have large wounded surfaces and they are neither ventilated nor perfused with blood. However, this also applies to parts of severely damaged lungs and other studies have shown that PCLS are a good model for innate immunity of the inflamed lung [35,36,37]. More research using living experimental animals and living *H. influenzae* strains are certainly needed to confirm these initial results.

Immune evasion strategies are the result of million years of co-evolution of microorganisms with their respective hosts and frequently, endogenous anti-inflammatory mechanisms are hijacked by well adapted pathogens [58,59]. Recently, we reported that dipalmitoyl-phosphatidylcholine, the most abundant lipid present in the pulmonary surfactant, efficiently inhibits inflammasome assembly and release of IL-1β by a mechanism that resembles that of PC-LOS [60]. A similar pathway is induced by the pulmonary anti-protease alpha1-antitrypsin [37]. The content of pulmonary dipalmitoyl-phosphatidylcholine is, however, reduced upon severe acute lung injury or inflammation [61] and alpha1-antitrypsin is consumed by proteases that are mainly released by activated neutrophils [62]. Hence, local inflammasome assembly and IL-1β release should be enabled in damaged lungs to allow efficient host defense. We speculate that PC-LOS of wild-type *H. influenzae* subverts the immune response of the host by preventing ATP-induced inflammasome assembly despite of a local lack in phosphatidylcholines and alpha1-antitrypsin.

The effect of PC-LOS is sensitive to nAChR antagonists targeting subunits α7, α9 and α10 that form an evolutionary conserved family of nAChR subunits [63]. Human bronchial epithelial cells express nAChR subunits α7 and α9 and respond with ion currents to nicotine or choline, known agonists of nAChRs [64]. We demonstrated before for human monocytic cells, that nicotine and free PC inhibit the BzATP-induced release of IL-1β via nAChR subunits α7, α9 and α10 [18,19]. Also, PC-LOS seems to signal via the same nAChR family. The data presented in this study are not unequivocal regarding the involvement of nAChR subunits α9 and/or α10. RgIA4 is an antagonist of nAChRs containing subunits α9 and/or α10 but cannot differentiate between both subunits [34]. However, as we demonstrated before, that nAChR subunits α7, α9 and α10 are needed for the PC-mediated metabotropic inhibition of monocytic P2X7 receptors [18,19,20] this might also hold true for pulmonary epithelial cells.

People carrying *H. influenzae* in their respiratory tract usually do not exhibit clinical signs of disease. Reduced mucociliary clearance, however, causes the propagation of *H. influenzae* resulting in otitis media, pharyngitis, bronchitis and pneumonia [65,66,67,68,69,70]. In patients suffering from chronic obstructive pulmonary disease (COPD), 50% of all exacerbations are caused by bacterial pathogens; among them, *H. influenzae* is most frequently isolated [70,71]. Treatment of COPD exacerbations includes antibiotics, corticosteroids and bronchodilators. Earl and colleagues showed that these regimens promote the persistence of *H. influenzae* and antibiotic resistance [72]. First antibiotic resistances to ampicillin and amoxicillin were described for *H. influenzae* as early as 1972. Later on, co-trimoxazole, combination of trimethoprim and sulfamethoxazole, became ineffective in the treatment of NTHi [73,74].

The solution to this problem could be a host centered anti-infective therapy beyond the classical antibiotic regimens that is less prone to the development of resistances. More precisely, we suggest that a short-term treatment of *H. influenzae*-colonized patients with antagonists of nAChRs might enable a vigorous inflammasome-dependent immune reaction and eradication of the infection.

In conclusion, we demonstrate that the ATP-induced release of IL-1β by pulmonary epithelial cells and by lung tissue (PCLS) is efficiently inhibited by PC-LOS from wild-type *H. influenzae* strains. PC-LOS signaling via archaic non-neuronal nAChRs seems to contribute to the immune evasion of *H. influenzae* and might be a promising therapeutic target.

## 4. Materials and Methods

### 4.1. Reagents

Mecamylamine hydrochloride, (−)-nicotine hydrogen tartrate salt (N5260), phosphocholine chloride calcium salt tetrahydrate, strychnine hydrochloride, and LPS (L2654 from *E. coli*) were obtained from Sigma-Aldrich (Taufkirchen, Germany). BzATP was provided by Jena Bioscience (Jena, Germany) and α-bungarotoxin by Tocris Bioscience (Bristol, UK). [V11L, V16D]ArIB and RgIA4 were produced and characterized as described previously [32,33,34]. Concentrations of all compounds used in this study were optimized in previous experiments on human monocytic U937 cells [18,19].

### 4.2. Purification and Characterization of PC-LOS

PC-LOS and PC-free LOS were isolated from various *H. influenzae* strains as described before [75]. In short, LOS was extracted from bacteria using a phenol/chloroform/light-petroleum method and further purified by ultracentrifugation. The structure of LOS was analyzed by high-field NMR and ESI-MS techniques. In addition, composition and linkage analyses were performed on *O*-deacylated LOS and oligosaccharide samples.

### 4.3. Pulmonary Epithelial Cell Lines

All cells were cultivated in a standard incubator at 37 °C, 5% CO_2_. The adherent human epithelial lung carcinoma cell line Calu-3 was obtained from the American Type Culture Collection (ATTC®, Manassas, VA, USA) and cultivated in ATCC-formulated Eagle’s MEM (No. 30-2003) supplemented with 10% fetal bovine serum (FBS, S-EUR30-I, Cell Concepts, Umkirch, Germany). A549 cells, adherent epithelial cells derived from a lung tumor, were provided by the Leibniz-Institute DSMZ (Braunschweig, Germany) and cultivated in Dulbecco’s MEM with GlutaMAX^TM^ (Gibco/Life Technologies, Carlsbad, CA, USA) containing 10% FBS. Epithelial cells were seeded at a density of 1 × 10^5^ cell in 1 ml medium in 12 well cell culture plates, cultivated for 24 h, followed by another 24 h stimulation with LPS (100 ng/mL). Epithelial cell cultures reached about 50% confluency. BzATP (100 µM) was added to the LPS-primed cells in the presence of different concentrations of nicotine, PC, PC-LOS or PC-free LOS. Thirty min later, cell-free cell culture supernatant was harvested and stored at −20 °C until measurement of IL-1β and LDH.

### 4.4. Mouse PCLS

Specified pathogen-free mice were obtained via Janvier Labs, Le Genest-Saint-Isle, France and were housed in our animal facility for about 2 weeks under a 12 h light/dark cycle and access to standard chow and water ad libitum. Experimental animals received humane care according to NIH “Guide for the Care and Use of Laboratory Animals”. The protocol was registered and approved by the local authorities (Regierungspräsidium Giessen, Germany; reference no. 571_M). Mouse PCLS were prepared using a modified protocol described previously [56,57,76]. Briefly, lungs were taken from 8 to 12 weeks old female C57BL/6NRj mice. The animals were sacrificed after anesthesia with isoflurane (5%); (Abbott, Wiesbaden, Germany) by cervical dislocation. The airways were filled via the cannulated trachea with 1.5% low melting agarose (Bio-Rad Medical Diagnostics, Dreieich, Germany) dissolved in HEPES-Ringer buffer. Lungs were removed and transferred to ice-cold HEPES-Ringer buffer to solidify the agarose. The lung lobes were cut into 350 µm thick slices using a vibratome (VT10000S, Leica, Bensheim, Germany). Slices were incubated in HEPES-Ringer buffer, supplemented with penicillin (100 U/mL, PAA, Etobicoke, Canada) and streptomycin (0.1 mg/mL, PAA) for at least 1.5 h at 37 °C to remove the agarose.

PCLS were washed once and cultured in Dulbecco’s modified Eagle’s medium (DMEM)/F-12 with L-glutamine and without HEPES (No. DF-042-B; Merck, Darmstadt, Germany) supplemented with 100 U/mL Pen Strep (Gibco) at 37 °C, 5% CO_2_, and 100% air humidity in 12-well tissue culture plates, using 2 slices per well. After 24 h of incubation, PCLS were treated with LPS (100 ng/mL), recombinant human IFN-γ (20 ng/mL; R&D Systems, Minneapolis, MN, USA) and recombinant human TNF-α (10 ng/mL; R&D Systems) for additional 24 h. As a control PCLS were left untreated for the same period of time. The functional integrity of PCLS produced according to the same protocol in the same laboratory was published recently [76]. Some of the untreated PCLS were fixed for 24 h in 4% paraformaldehyde (Carl Roth, Karlsruhe, Germany) and lightly stained with Richardson’s blue as described before [77]. Thereafter, PCLS were stimulated for 30 min with BzATP (150 µM) in presence and absence of RMPC (1 µg/mL) or NTHiPC (1 µg/mL). Tissue culture supernatants were harvested and stored at −20 °C until measurement of IL-1β and LDH

### 4.5. Cytokine Measurement

IL-1β was measured in cell-free cell culture supernatants by the human IL-1 beta/IL-1F2 DuoSet® ELISA (R&D Systems, Minneapolis, MN, USA) or mouse Quantikine® IL-1β Immunoassay (R&D Systems) according to the instructions of the manufacturer. IL-1β values of PCLS supernatants were normalized to the total protein content of the tissue slices that was assessed using the Micro BCA Protein Assay Kit (Thermo Fisher Scientific, Waltham, MA, USA).

### 4.6. LDH Measurement

LDH was measured in cell culture supernatants by the Non-Radioactive Cytotoxicity Assay (Promega, Madison, WI, USA) according to the manufacturer’s instructions. The proportion of dead cells was estimated by including a maximum LDH release control. For this purpose, an equivalent number of A459 or Calu-3 cells were lyzed by two cycles of freezing (−80 °C) and thawing. The amount of LDH in this control sample was set to 100% and the relative cell death in all other experiments was calculated accordingly.

### 4.7. Statistical Analyses

Data were analyzed with the IBM SPSS Statistics software Version 25 (IBM, Munich, Germany). Values derived from different cells were compared, where applicable, by the non-parametric Kruskal-Wallis test, followed by the Mann-Whitney rank-sum test. The Wilcoxon signed-rank test was used for analyses of dependent values. Data were visualized using program Inkscape version 0.92 (Free and Open Source Software licensed under the GPL). The number (n) of individual experiments is indicated in the Results section and the Figures.
